# Chloroform

**Published:** 1866-07

**Authors:** 


Chloroform.—The frequent fatal accidents from the administration of chloroform have had this result that the use of this anæsthetic is gradually being discarded by the profession. In Lyons it has not been used for years, and the medical society of that city has after long discussion, adopted unanimously the following conclusions:

1. Rectified ether employed as an anæsthetic is less dangerous than chloroform. 2. Anæsthesia is produced as regularly and completely by rectified ether as by chloroform. 3. Ether is in some respects less convenient in use than chloroform; but the inconveniences attending its use are of little consequence when compared with the dangers inherent in the employment of chloroform. 4. Consequently, ether should be preferred to chloroform.

